# Prospective Comparison of Longer Needle Lengths to Assess the Risk of OnabotulinumtoxinA-Associated Neck Pain in Patients with Chronic Migraine

**DOI:** 10.3390/toxins14070434

**Published:** 2022-06-24

**Authors:** Emmanouil V. Dermitzakis, Michail Vikelis, George S. Vlachos, Andreas A. Argyriou

**Affiliations:** 1 Euromedica General Clinic, 54645 Thessaloniki, Greece; manolis.dermitzakis@gmail.com; 2 Headache Clinic, Mediterraneo Hospital, 16675 Glyfada, Greece; mvikelis@headaches.gr (M.V.); gvlachos@neuromed.gr (G.S.V.)

**Keywords:** OnabotulinumtoxinA, BoNTA, chronic migraine, tolerability/safety, neck pain, Body Mass Index, needle length

## Abstract

Background: We aimed to prospectively assess the role of needle length in improving the tolerability/safety profile of OnabotulinumtoxinA (BoNTA) for chronic migraine (CM) prophylaxis, with a specific focus on neck pain, based on patients’ body habitus and other variables. Methods: BoNTA was administered quarterly for two consecutive cycles, using the standard 0.5-inch 27 G needle at all pre-defined PREEMPT injection sites, except the left-hand side trapezius and paraspinal muscles, which were injected using longer needles of 1-inch 23 G at first and 1-inch 27 G at second infusion. Participants were interviewed at day 14 following each session for evidence of neck pain. The predictive significance of Body Mass Index (BMI) and other variables with neck pain was also examined. Results: A total of 100 consecutive CM patients were evaluated, and each patient served as her/his self-control. The incidence, duration and intensity of neck pain did not significantly differ using either 1-inch needle compared with standard 0.5-inch 27 G needle, although the incidence and characteristics of neck pain with the use of longer needles appeared slightly higher and more intense. The BMI index and other tested variables remained unrelated to neck pain. Conclusions: We were not able to identify significant differences or correlations in the incidence, characteristics and location of neck pain with the use of different needle length to inject BoNTA, although the use of the longer 1-inch needles likely increased the risk of this adverse event.

## 1. Introduction

Migraine appears to be the seventh most common disease worldwide, with an estimated global prevalence of 15.3% [1]. Based on migraine’s attack frequency, it can be divided into two forms, i.e., chronic or episodic migraine [2]. According to epidemiological estimates, about 2.5% of migraineurs will progress each year from episodic to chronic migraine (CM), with the latter being defined as headaches occurring on at least 15 days per month, with eight of these having typical migrainous features for more than 3 months [3]. There is an obvious clinical need to effectively manage CM by preventing migraine chronification and by inhibiting central sensitization with the use of effective preventative pharmacological agents, because patients with CM experience severe pain intensity, greater disability and much reduced productivity at work/school than patients with episodic migraine [3,4].

OnabotulinumtoxinA (BoNTA) stands worldwide among the established treatment options for CM prophylaxis, following the publication of pooled results from the double-blind, randomized, placebo-controlled phases of the PREEMPT (Phase III REsearch Evaluating Migraine Prophylaxis Therapy) clinical program [5]. These pooled results demonstrated a favorable benefit–risk ratio of BoNTA when commenced in CM patients with or without medication overuse headache (MOH). Moreover, recent evidence from long-term experience of BoNTA in CM patients for up to five years of treatment supports its sustained efficacy/safety as well as tolerability over time in addition to demonstrating considerably increased adherence coupled with improvements in patient-reported outcomes [6]. BoNTA has been approved for CM prophylaxis in Greece according to both international [7] and national Greek guidelines [8]; it is reimbursed by the Greek National Health System and social services as a third-line preventive treatment option in patients having failed or not tolerated two previous oral prophylactic medications of first choice for CM.

The role of patients’ body habitus and its potential impact on tolerability profile of BoNTA injections for the prevention of migraines has been minimally explored. Wu-Fienberg et al. [9] were the first to evaluate the relationship between BMI and the distance from skin to muscle fascia at various BoNTA CM injections sites.

The results showed that the temporalis and trapezius muscles lie deeper than can be reached using the standard 0.5-inch needle in severely obese (BMI > 35 kg/m^2^) patients. In addition, the semispinalis capitis muscle lay deeper than can be reached using the standard 0.5-inch needle in both the obese (BMI 30–35 kg/m^2^) and the severely obese (BMI > 35 kg/m^2^) patients of the study population. Thus, a 0.5-inch needle is insufficient to allow intramuscular BoNTA injection into the trapezius and semispinalis capitis muscle in a majority of overweight and obese patients [9].

Previous experience from vaccines indicates that serious reactions to intramuscular injections are rare. However, subcutaneous injections can cause an increased incidence of local reactions, such as irritation, inflammation and granuloma formation. These reactions are attributed to the fact that adipose tissue has much poorer drainage channels and subsequently retains injected materials [10]. To make sure the needle does not seep into subcutaneous tissue, the decision on the size of the needle should be made individually for each person.

Given that obesity is associated with increased migraine prevalence, attack frequency, and disability, a patient’s BMI may be important for treatment planning in order to ensure effective and well-tolerated intramuscular uptake of BoNTA [9]. That said, clinical practice needs to reflect considerations about the appropriate length of needles. In this work, we sought to identify any observable changes in the tolerability profile of BoNTA-treated CM patients when different needle lengths are used. Particularly, mainly taking under consideration Body Mass Index (BMI), our primary aim was to compare different needle lengths to assess the risk of BoNTA-associated neck pain in patients with CM, which is considered to be the most common adverse event (up to 9%) to occur after BoNTA application [5]. In addition, we also sought to assess the patients’ self-perceived impact of disease management and satisfaction from BoNTA treatment with the use of longer needles.

## 2. Results

A total of 100 consecutive CM patients, who according to standard clinical practice were scheduled for BoNTA treatment, were initially enrolled, and all (100%) of them achieved treatment with the 2nd BoNTA course, according to the study’s schedule. Each of these 100 patients served as her/his self-control, and no additional patients were used for comparison. Patients were treated quarterly with BoNTA, strictly adhering to the fixed-dose, fixed-sites PREEMPT paradigm [5] and using the standard 0.5-inch 27 G needle, with the exception of the left-hand side trapezius and paraspinal muscles, in which different needle lengths and gauges were used, as explained in detail in the material and methods section. Afterwards, safety/tolerability outcomes were compared between right= vs. left-hand side trapezius and paraspinal muscles, as described at the last section of the text.

The study sample consisted of 14 males (14%) and 86 females (86%) with a mean age of 44.5 ± 10.3 (range: 19–60) years. Patients had failed a median number of three (range: 1–7) previous oral prophylactic medications. A total of 18/100 (18%) enrolled patients were BoNTA naïve, with the remaining 82 patients previously exposed to a median of four (range: 1–16) BoNTA quarterly administered sessions. Among these 82 pre-treated patients, six (7%) reported neck pain during their previous exposure to BoNTA therapy. Psychiatric comorbidities were common, being evident in 69/100 patients (69%). Of those 69 patients, 24 (34.8%) had anxiety disorder; 22 (31.9%) had depression; 20 (29%) were diagnosed with mixed anxiety and depression disorder; and 3 (4.3%) had bipolar disorder. Table 1 describes in detail the baseline epidemiological and clinical characteristics of participants, including the classification according to their BMI status.

### 2.1. Incidence and Characteristics of Neck Pain according to Needle Length

**Figure 1:**
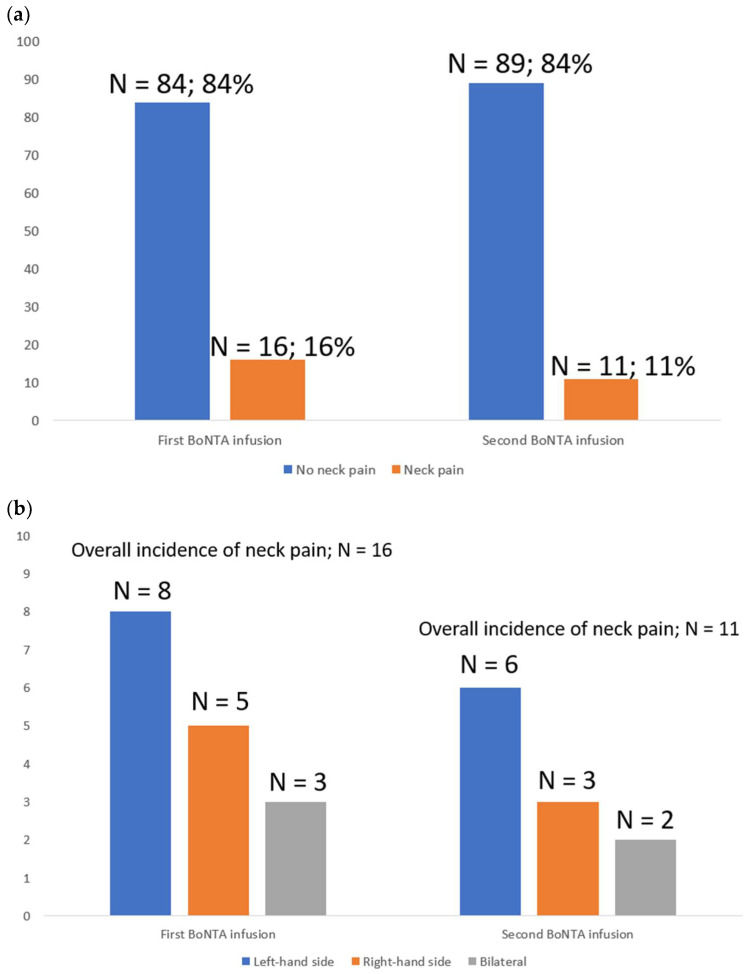
(**a**) Incidence of neck pain after BoNTA infusions and (**b**) depicts the location of neck pain after BoNTA infusions.

At first BoNTA administration, the use of a 1-inch 23 G needle in the left-hand side trapezius and paraspinal muscles resulted in 16 (16%) patients reporting neck pain: 8 at left, 3 at right and 5 bilateral. At second repeated infusion using the 1-inch 27 G needle to inject BoNTA in the left-hand side trapezius and paraspinal muscles, there were 11 (11%) patients reporting neck pain: 6 at left, 3 at right and 2 bilateral. The difference in the incidence and location of neck pain between the first and second BoNTA infusion remained insignificant (Figure 1a,b). Figure 1a refers to the number and percentage (N; %) of patients with evidence of neck pain at both first and second BoNTA infusions out of the 100 initially enrolled patients. Figure 1b depicts the location of neck pain in those 16 and 11 patients experiencing this BoNTA-related adverse event after both first and second infusions, respectively.

**Figure 2:**
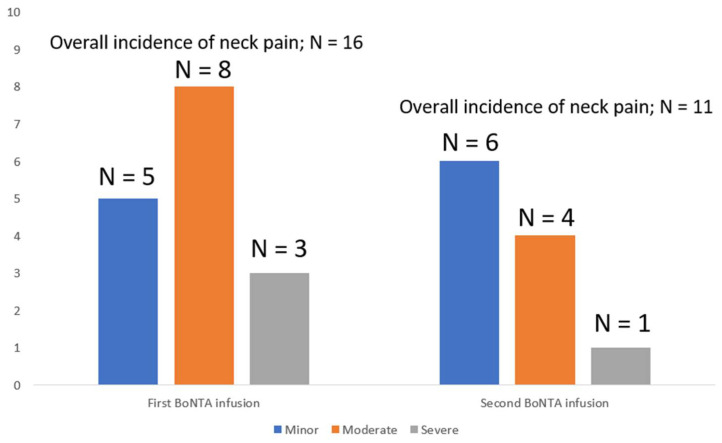
Intensity of neck pian after BoNTA infusions.

The duration of neck pain without taking into account the side of the neck pain was 7.1 ± 6.7 (range: 1–14) days, while at second BoNTA infusion, the duration was shorter than that observed at the first infusion, but without reaching significance (4.5 ± 4.4 (range: 1–14) vs. 7.1 ± 6.7 (range: 1–14); *p* = 0.09) days. Similar to the first infusion, the left-hand side vs. other side neck pain was slightly higher in duration at the second infusion with the use of a 1-inch 27 G needle. The reported severity of neck pain was comparable between the first and second BoNTA infusions, being rated at first infusion as minor in five patients, moderate in eight and severe enough to require prescription of NSAIDs in three patients vs. minor (*n* = 6), moderate (*n* = 4) and severe (*n* = 1) at phone interviewing conducted three weeks after the second BoNTA course (Figure 2).

**Figure 3:**
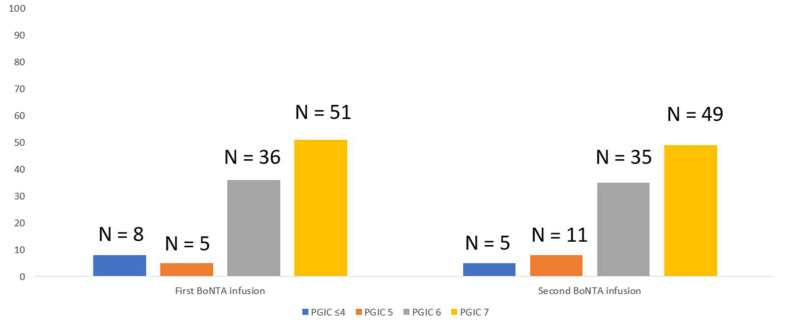
Satisfaction of participants (n = 100) to BoNTA therapy, according to PGIC measure.

In any case, the duration and severity of neck pain at first infusion influenced the BoNTA-attributed perception of change and satisfaction of affected patients, as 8 of 11 patients experiencing moderate/severe intensity remained dissatisfied with a score ≤ 4 on “Patient Global Impression of Change” (PGIC). The remaining 92 patients reported an at least clinically meaningful benefit (PGIC ≥ 5) with the first BoNTA treatment course. At second BoNTA infusion, all five patients experiencing moderate/severe intensity scored PGIC ≤ 4 to express their dislike. It is worth noting that rating PGIC ≤ 4 after both the first and second BoNTA infusion attributed to moderate/severe neck pain remained unrelated (spearman’s rho: 0.363; *p* = 0.6) to the clinical benefit experienced by patients, as this was expressed by the reduction in mean migraine days. PGIC scorings after both first and second BoNTA infusions are shown in Figure 3.

### 2.2. Direct Comparison and Associations of Neck Pain and Different Needle Lengths

There was no significant difference in the location of neck pain (left-hand side vs. other) either with the use of the 1-inch 23 G needle (*p* = 0.1) to inject BoNTA or when using the 1-inch 27 G needle (*p* = 0.74). The logistic regression analysis failed to identify predictors being able to independently predict the manifestation of neck pain at either of the two BoNTA infusions. Specifically, gender (*p* = 0.335 with 1-inch 23 G needle and *p* = 0.676 with 1-inch 27 G needle); age (*p* = 0.284 with 1-inch 23 G needle and *p* = 0.433 with 1-inch 27 G needle); BMI (*p* = 0.611 with 1-inch 23 G needle and *p* = 0.851 with 1-inch 27 G needle); the number of previous BoNTA cycles in pre-treated patients (*p* = 0.483 with 1-inch 23 G needle and *p* = 0.452 with 1-inch 27 G needle) and the evidence of neck pain during previous exposure to BoNTA therapy (*p* = 0.393 with 1-inch 23 G needle and *p* = 0.576 with 1-inch 27 G needle) failed to significantly correlate with the left-hand side vs. other location of neck pain.

## 3. Discussion

The fixed site-fixed dose strategy, according to the PREEMPT paradigm, pointing at the peripheral nerve endings of the cervical and trigeminal sensory system, has been widely used in the prophylactic treatment of CM with BoNTA. Clinical practice shows that adherence to this treatment paradigm is associated with efficacy and minimization of adverse events. Indeed, a recent metanalysis of available randomized, double-blind, placebo-controlled trials, injecting BoNTA as per protocol, has demonstrated that BoNTA is superior to a placebo with only few and mild adverse events in the clinical setting of CM prophylaxis. Nonetheless, clinical practice shows that even if BoNTA is administered by an experienced injector, patients are still at risk of developing side effects in the cervical paraspinal muscle group, including the trapezius, splenius capitis and cervicis, and semispinalis capitus, with neck pain standing among the most frequent relevant complications. Existing knowledge supports that neck pain, although usually transient and self-limiting soon after BoNTA is metabolized in the different areas of the head and neck, can be notable in some patients [11].

Nonetheless, little attention has been specifically paid, thus far, to the effect of patients’ body habitus and other variables upon the adverse event profile of BoNTA for preventing CM. Using at first infusion a 1-inch 23 G needle to inject BoNTA at the left-hand side paraspinal and trapezius muscles and at second infusion a 1-inch 27 G needle, instead of the standard 0.5-inch 27 G needle, we sought to herein provide some insights on the role of needle length in improving the tolerability/safety profile of BoNTA, emphasizing neck pain and its relation to BMI and other demographic information as clinical factors. Our participants reported similar incidence of neck pain with the use of different needle lengths. Likewise, the duration and intensity of neck pain did not significantly differ using either the 1-inch 23 G needle or the 1-inch 27 G needle, compared with the standard 0.5-inch 27 G needle. Our observation on neck pain incidence with the standard 0.5-inch 27 G needle (up to 5%) is generally in line with previous studies [12]. It should be noted that, without reaching statistical significance, the incidence of neck pain with the 1-inch needles was slightly higher and pain was more intense, likely because of potential damage to deeper cervical paraspinal areas with the use of longer needles. This finding advocates in favor of the previously discussed view, suggesting that BoNTA injections into the trapezius (for a total of 30 units divided across six sites) and paraspinal muscles (for a total of 20 units divided across four sites) should be superficially administered, according to the PREEMPT injection paradigm: angling the needle 45° and superiorly. Moreover, it is advised that patients should be positioned in a proper way to avoid injecting BoNTA too deep or below the hairline in mid and lower cervical regions in order to minimize the risk of neck pain and/or neck weakness [13,14].

It is generally considered that patients with preexisting neck pain might be more liable to experience neck pain exacerbation after BoNTA injection in the cervical paraspinal muscle group, whereas patients of smaller frames may be also more prone to neck pain and weakness [14]. Neither of the latter two relationships was seen in the present study. Particularly, BMI index remained unrelated to the incidence and characteristics of neck pain with the use of 1-inch needles, contrary to recent evidence of an imaging study suggesting that the use of longer needles may result in more effective intramuscular uptake to facilitate improved BoNTA response [9]. As such, the use of different needles, according to individualized patient body habitus, should be cautiously considered when deciding the treatment plan, because the strategy to use longer needles may enhance BoNTA efficacy but at the same time may also compromise its safety/tolerability profile with evidence of neck pain.

Our study has some limitations, which are related to its observational design and might relate to the relatively overall small sample size and the corresponding low numbers of patients experiencing neck pain, likely resulting in insufficient power to detect medium effect sizes. However, provided that this is a self-controlled study, because each patient serves as her/his own control, we might suggest that we were able to produce results that are statistically and clinically valid with far fewer patients than would otherwise be required; a similar outcome is expected to occur with the use of larger sample size.

Finally, it is important to clarify that longer needles would under no circumstances result in PREEMPT protocol violation, as based on the available data they would result in injecting the toxin deeper within the selected muscle (for the trapezius muscles of patients with BMI < 35 kg/m^2^ and semispinalis capitis muscle of patients with BMI of <30 kg/m^2^) or injecting the toxin to the belly of the selected muscle (for the trapezius muscles of patients with BMI > 35 kg/m^2^ and semispinalis capitis muscle of patients with BMI of 30–35 and >35 kg/m^2^). More specifically, the length of the used needle at the left-hand side trapezius and paraspinal muscles was 1-inch, which converts into 25.4 mm, and the depth of the muscles under investigation is considerably lower based on measurements of the median muscle depth with the use of computed tomography scans of the head and neck [9]. Hence, the use the shorter needles would result in subcutaneous rather than intramuscular BoNTA injections, thoroughly increasing the risk for painful local reactions [10].

## 4. Conclusions

To conclude, we were not able to identify significant differences in the incidence, characteristics and location of neck pain with the use of longer needle length to inject botulinum toxin type A subtype A1 [15]. On clinical grounds, BMI index and other confounding variables, e.g., age and gender, remained unrelated to neck pain, although the use of longer needles of 1-inch likely increased the risk of this adverse event. Nonetheless, we might suggest that our study provided some meaningful insights into the role of needle length in improving the tolerability/safety profile of BoNTA for CM prophylaxis. Longer needles should cautiously be used to inject BoNTA for CM prophylaxis, as unwanted outcomes could occur. A superficial approach to inject BoNTA in the cervical paraspinal muscle group with the use of the standard 0.5-inch needles might be suitable for the majority of treated patients to achieve both adequate efficacy and tolerability/safety profile, as previously recommended after reviewing the method of BoNTA injection for chronic migraine, based on the PREEMPT clinical program [16].

## 5. Materials and Methods

This was a prospective three-center study. Patients were included in this study if they (i) were over 18 years of age at enrolment (ii) had been diagnosed with CM with or without medication overuse (iii) were either BoNTA treatment-naïve or treatment-experienced, (iv) agreed to keep headache diaries as per their treating physicians’ instructions and (v) were consistent in conducting phone interviews according to the study needs. Otherwise, general inclusion and exclusion criteria were the same as previously described [6,17,18].

For the purposes of the current study, eligible patients were classified at baseline as normal weight (BMI < 25 kg/m^2^), overweight (BMI 25–30 kg/m^2^), obese (BMI 30–35 kg/m^2^) and severely obese (BMI > 35 kg/m^2^) based on their BMI index [9]. Initial screening also involved recording of demographic and migraine clinical information, review of medical history, physical examination and BoNTA safety experience in pre-treated patients. Treatment with BoNTA (Botox^®^ 100UI/fl, Allergan-Abbvie, Hellas) was administered quarterly from certified BoNTA injectors throughout the study’s period, according to the PREEMPT paradigm [5]. In total, patients received 2 treatment cycles, and subsequently the study duration was 6 months.

As can be seen in Table 2, at first infusion, enrolled patients received BoNTA using the standard 0.5-inch 27 G needle at the pre-defined PREEMPT injection sites of procerus, corrugator, frontalis, temporalis and occipitalis muscles as well as the trapezius and paraspinal muscles on the right-hand side. On the left-hand side, the trapezius for a total of 15 units divided across three sites and paraspinal muscles for a total of 10 units divided across two sites were specifically injected using a 1-inch 23 G needle, whereas the standard 0.5-inch 27 G needle was used to inject BoNTA at the remaining pre-defined sites. At the second infusion 3 months afterward, the pre-defined sites on the right-hand side were injected with the standard 0.5-inch 27 G needle, whereas on the left-hand side the trapezius and paraspinal muscles were specifically injected using a 1-inch 27 G needle, with the remaining pre-defined sites injected with the standard 0.5-inch 27 G needle. At day 14 following the first and second BoNTA infusions, participants were contacted by phone, and any AEs, including neck pain, muscle spasm and inflammation, were recorded per left vs. right-hand side when they were re-contacted at day 30 after both infusions to provide data about BoNTA efficacy, i.e., change in monthly migraine days, based on their headache questionnaires.

The overall patients’ self-perceived impact of disease management and satisfaction from BoNTA treatment after each scheduled infusion was assessed using the short self-report 7-point patients’ reported outcome (PRO) questionnaire PGIC, where 1 stands for “no change” and 7 for “considerable improvement” [19]. A cut-off of PGIC ≥ 5 was set to define patients experiencing a “clinically meaningful benefit” with BoNTA treatment, according to the IMMPACT recommendations [20].

### Statistical Analysis

Descriptive statistics were generated for all variables, depending on the nature of the variable, using the SPSS for Windows (release 27.0; SPSS Inc., Chicago, IL, USA). Non-parametric statistics using the Kruskall–Wallis one-way analysis of variance were computed to ascertain potential differences in the occurrence of left- vs. right-hand side neck pain at each of the two consequent BoNTA infusions. A linear regression analysis using the left-side neck pain as the dependent variable was performed to identify predictors of its occurrence. All tests were two-sided, unless otherwise stated, and significance was set at *p* < 0.05.

## Figures and Tables

**Table 1:** Demographic and clinical characteristics of participants.

Variable	Participants	
	*n = 100*	
	N	%
**Gender**		
females	86	86
males	14	14
**Age ± SD (range)**	44.5 ± 10.3 (19–60)	
**Previous lines of prophylactic medications** Median value (range)	3 (1–7)	
**Years ± SD (range) with chronic migraine**	10.6 ± 3.5 (6–18)	
**Psychiatric comorbidities**	69	69
**BoNTA experience**		
Naïve	18	18
Pre-treated	82	82
**BMI**		
Normal (BMI < 25 kg/m^2^)	60	60
Overweight (BMI 25–30 kg/m^2^)	22	22
Obese (BMI 30–35 kg/m^2^)	15	15
Severely Obese (BMI > 35 kg/m^2^)	3	3

**Table 2:** Template of comparing needle sizes according to right-hand vs. left-hand side. Each patient served as her/his self-control.

	Needle Used at 1st Infusion	Needle Used at 2nd Infusion
*Right-hand side trapezius and paraspinal muscles and all other PREEMPT protocol sites in the forehead and temporalis *	Standard 0.5-inch 27 G	Standard 0.5-inch 27 G
*Left-hand side trapezius and paraspinal muscles*	1-inch 23 G	1-inch 27 G

## Data Availability

The data that support the findings of this study are available from the corresponding author upon reasonable request.
